# Docosahexaenoic acid-containing choline phospholipid modulates LPS-induced neuroinflammation in vivo and in microglia in vitro

**DOI:** 10.1186/s12974-017-0939-x

**Published:** 2017-08-24

**Authors:** Célia Fourrier, Julie Remus-Borel, Andrew D. Greenhalgh, Michel Guichardant, Nathalie Bernoud-Hubac, Michel Lagarde, Corinne Joffre, Sophie Layé

**Affiliations:** 1 INRA, Nutrition et Neurobiologie Intégrée, UMR 1286, 33076 Bordeaux, France; 2 Bordeaux University, Nutrition et Neurobiologie Intégrée, UMR 1286, 33076 Bordeaux, France; 3 CarMeN laboratory, INSERM UMR 1060, INRA UMR 1397, IMBL, INSA-Lyon, University of Lyon, Lyon, France

**Keywords:** DHA, AceDoPC, Microglia, IL-6, STAT3, Intravenous, TNFα, IL-1β, Phospholipid, PC-DHA

## Abstract

**Background:**

Neuroinflammatory processes are considered a double-edged sword, having both protective and detrimental effects in the brain. Microglia, the brain’s resident innate immune cells, are a key component of neuroinflammatory response. There is a growing interest in developing drugs to target microglia and control neuroinflammatory processes. In this regard, docosahexaenoic acid (DHA), the brain’s n-3 polyunsaturated fatty acid, is a promising molecule to regulate pro-inflammatory microglia and cytokine production. Several works reported that the bioavailability of DHA to the brain is higher when DHA is acylated to phospholipid. In this work, we analyzed the anti-inflammatory activity of DHA-phospholipid, either acetylated at the sn-1 position (AceDoPC, a stable form thought to have superior access to the brain) or acylated with palmitic acid at the sn-1 position (PC-DHA) using a lipopolysaccharide (LPS)-induced neuroinflammation model both in vitro and in vivo.

**Methods:**

In vivo, adult C57Bl6/J mice were injected intravenously (i.v.) with either AceDoPC or PC-DHA 24 h prior to LPS (i.p.). For in vitro studies, immortalized murine microglia cells BV-2 were co-incubated with DHA forms and LPS. AceDoPC and PC-DHA effect on brain or BV-2 PUFA content was assessed by gas chromatography. LPS-induced pro-inflammatory cytokines interleukin IL-1β, IL-6, and tumor necrosis factor (TNF) α production were measured by quantitative PCR (qPCR) or multiplex. IL-6 receptors and associated signaling pathway STAT3 were assessed by FACS analysis and western-blot in vitro.

**Results:**

In vivo, a single injection of AceDoPC or PC-DHA decreased LPS-induced IL-6 production in the hippocampus of mice. This effect could be linked to their direct effect on microglia, as revealed in vitro. In addition, AceDoPC or PC-DHA reduced IL-6 receptor while only AceDoPC decreased IL-6-induced STAT3 phosphorylation.

**Conclusions:**

These results highlight the potency of administered DHA—acetylated to phospholipids—to rapidly regulate LPS-induced neuroinflammatory processes through their effect on microglia. In particular, both IL-6 production and signaling are targeted by AceDoPC in microglia.

## Background

Docosahexaenoic acid (DHA) is a prominent long chain omega-3 polyunsaturated fatty acid (LC PUFA) of the brain, where it regulates both the structure and function of neurons, glia, and endothelial cells [1]. This PUFA also exerts protective activity against inflammation in the brain. Indeed, using a combination of dietary and transgenic mice models, we and others have demonstrated that increased brain DHA attenuates neuroinflammatory processes, while decreased brain DHA promotes them [2–10]. DHA’s role in neuroinflammation is likely due to its direct effect on microglia, the resident innate immune cells of the brain, and its oxygenase-derived metabolites (neuroprotectins, resolvins, maresins) and endocannabinoid-like metabolites [2, 9, 11–18]**.**

Brain DHA levels rely on dietary content [19]. DHA is either synthesized from the essential PUFA alpha-linolenic acid (ALA, 18:3 n-3) or consumed preformed from marine sources [20, 21]. Despite the brain expressing the enzymes necessary to synthesize DHA from ALA, the synthesis rate is much lower than the rate of DHA uptake from the plasma, suggesting that the diet is a primary source of DHA to the brain [22–24]. Once ingested, DHA is rapidly redistributed within (1) plasma lipoproteins, mainly in triacylglycerols (TG) or phospholipids (PL) (which constitute the major destination for DHA) and (2) the albumin fraction as non-esterified DHA or lysophosphatidylcholine-esterified DHA (lysoPC-DHA) in the blood [25–27].

Several works report that the bioavailability of DHA to the brain is better when DHA is in its lysoPC form in the blood [28–31]. However, how DHA enters the brain is still a matter of debate as both unesterified and lysoPC-DHA forms are good source of brain DHA [26, 32]. Several reports are in favor of a preferential crossing of the blood-brain barrier (BBB) when DHA is esterified at the sn-2 position of lysoPC-DHA [28, 31]. This is supported by the recent discovery of MFSD2A (major facilitating superfamily domain-containing protein 2A), an exclusive transporter of lysoPC-DHA expressed by endothelial cells of BBB [33]. Importantly, in regard to brain diseases, DHA in the form of lysophospholipid has a longer plasma half-life and, as a result, increases DHA brain exposure [26].

In the lysoPC form, DHA is initially esterified at the sn-2 position. However, it rapidly changes from the physiological sn-2 position to the more stable but non-physiological sn-1 position. Then, to prevent such a migration, we acetylated the sn-1 position that results in a structured phospholipid, 1-acetyl,2-docosahexaenoyl-PC (AceDoPC) [34]. This structure mimics and behaves like 2-DHA-lysoPC in terms of DHA accumulation in the brain and has been shown to exert different biological properties such as the prevention of experimental stroke or the inhibition of the platelet-activating factor-induced aggregation in rats [34, 35].

Despite the possibility of a privileged entry of AceDoPC in the brain [36], its potential effect on neuroinflammatory pathways has not been explored. In this work, we investigated the effects of AceDoPC as compared to PC-DHA(1-palmitoyl,2-docosahexaenoyl-PC), previously shown to also increase delivery of DHA to brain when acutely administered [4]. We tested this in lipopolysaccharide (LPS)-induced neuroinflammation in both in vitro and in vivo models and probed the underlying signaling mechanisms. We found that a single in vivo administration of AceDoPC or PC-DHA specifically reduced LPS-induced interleukin-6 (IL-6), a pro-inflammatory cytokine, in the hippocampus but not in the hypothalamus. In vitro, AceDoPC significantly decreased microglia activation and IL-6-induced nuclear translocation of STAT3 in microglia cells through the downregulation of IL-6 receptor subunit gp130. AceDoPC effect on microglia and neuroinflammatory pathways suggests a potential therapeutic avenue for inflammation-related brain diseases.

## Methods

### Animals and treatments

Animal husbandry and experimental procedures were in accordance with the EU Directive 2010/63/EU for animal experiments and approved by the national ethical committee for care and use of animals (approval ID A13169). C57BL6/J mice from Janvier (Le Genest St Isle, France) were received at 10 weeks of age, housed individually and maintained in a temperature (22 ± 1 °C) and humidity controlled facility on a 12 h light-dark cycle with free access to food and water. They were fed a standard chow diet (A04, Safe, Augy, France). Experiments were performed at 12 weeks of age. The day before the experiment, plasma from C57BL/6 J (*n* = 10) was isolated as previously described [35]. Briefly, mouse blood was collected in tubes containing heparin and centrifuged for 10 min at 2600 g. Plasma was collected and incubated overnight at 4 °C with AceDoPC [34] or PC-DHA [34] or vehicle. Then, the mice were injected intravenously in the caudal vein at the following concentrations: 3.23 μg/g of mouse for AceDoPC and 4.33 μg/g of mouse for PC-DHA. Doses of AceDoPC and PC-DHA administrated are equivalent to 1.7 μg/mouse or ≈ 66 μM/mouse of DHA/mouse. Twenty-four hours after injection, mice were injected intraperitoneally with LPS (*Escherichia coli* 0127:B8, 500 μg/kg) or NaCl (*n* = 3–6 per group) [20, 37]. Mice were sacrificed 6 h later by decapitation after isoflurane anesthesia. Hippocampus and hypothalamus were quickly collected, frozen on dry ice and stored at −80 °C until analysis. Blood samples were obtained by cardiac puncture and collected in tubes containing 10% EDTA. After 10 min centrifugation (10,000 g, 4 °C), plasma was collected and kept at −80 °C.

### Cell culture and treatments

BV-2 cells, an immortalized murine microglial cell line, were cultured as previously described [16] in RPMI medium (Invitrogen, Carlsbad, CA, USA) supplemented with 10% heat-inactivated fetal calf serum (Eurobio, Courtaboeuf, France), streptomycin sulfate (50 μg/mL), phenoxypenicillinic acid (65 μg/mL), and glutamine (65 μg/mL) in 5% CO_2_ at 37 °C. Cells were seeded at a density of 500,000 cells/well in 6-well culture plates. When cells reached 75% confluency, they were serum-starved for 24 h in RPMI. Then, they were treated for 24 h in serum-free medium containing 50 μmol/L fatty acid-free bovine serum albumin (BSA, Sigma Aldrich) added with PC-DHA (30 μM) or AceDoPC (30 μM) in 0.1% ethanol or 0.1% ethanol used as vehicle. These concentrations were chosen on the basis of optimal biological effects of DHA previously reported [2]. In addition, the solvent used (0.1% ethanol) has no effect on cytokine production and cell viability [2, 16]. Where specified, cells were further treated with 1 μg/mL LPS (*E. coli*, 0127:B8, Sigma Aldrich, Lyon, France) for 3 or 6 h or with 10 ng/mL interleukin-6 (IL-6, R&D Systems, Lille, France) diluted in NaCl for 30, 60, or 90 min. BV-2 cells treated with NaCl alone were used as control [2, 16].

### Plasma cytokine assay

Cytokine assays were performed as previously described [8]. The limit of detection was 1.1 pg/ml for IL-6, 2.3 pg/ml for TNFα, 5.4 pg/ml for IL-1β, 1.1 pg/mL for IFNγ, and 2.0 pg/mL for IL-10. Briefly, samples diluted 1/2 were added to a 96-well microtiter plate (25 μL/well) coated with beads (Millipore, France), covered with aluminum foil, and incubated overnight on a shaker at 4 °C in the dark. After removal of sample using a magnet, beads were incubated with detection antibodies for 1 h at room temperature while shaking, followed by streptavidin-PE for 30 min. The beads were then re-suspended in 150 μL sheath fluid and analyzed using the BioPlex 200 system (Bio-Rad, France). The reader was set to read a minimum of 50 beads with an identical fluorescence expressed as the median fluorescence intensity. Median fluorescence intensity readings were converted to pg/mL using calibration curves prepared with cytokine standards included in the kit.

### RT-PCR

Total RNA were extracted from brain structures and BV-2 cells using Trizol (Invitrogen, Life Technologies). RNA purity and concentration were determined using a Nanodrop spectrophotometer (Nanodrop technologies, Wilmington, DE). One microgram of RNA was reverse transcribed to synthesize cDNA using Superscript III and oligo random hexamers (Invitrogen, Life Technologies™, Saint-Aubin, France) [5]. Quantitative PCR was then performed using the Applied Biosystems Assay-on-Demand Gene Expression Products protocol (Foster City, CA, USA), as previously described [6]. Briefly, target cDNAs and reference cDNAs (β2-microglobulin) were amplified by PCR using Taqman gene expression assays for sequence-specific primers purchased from Applied Biosystems (Foster city, CA, USA) [3, 5, 38]. We focused on the expression levels of IL-1β, IL-6, and TNFα as pro-inflammatory markers. Reactions were performed in duplicate according to manufacturer’s instructions as previously described [39]. PCR program consisted of 40 cycles of 95 °C for 15 s and 60 °C for 1 min. Fluorescence was measured using an AB 7500 Real-Time PCR system (Applied Biosystems, Foster city, CA), and final quantification was carried out using the comparative threshold (Ct) method as previously described [3, 6, 7, 40]. Results are expressed as relative fold change to control target mRNA expression.

### FACS analysis

BV-2 cells preparation was incubated with anti-CD16/CD32 antibody (rat, eBiosciences, Paris, France) to block Fc receptors for 5 min on ice. Cells were washed and then incubated for 1 h with anti-gp80-PE (rat, Biolegend, Ozyme, France) and anti-gp130-APC (rat, eBiosciences, Paris, France). After washing, cells were then suspended in PBS/BSA 0.1% for analysis. Non-specific binding was assessed by using non-specific, isotype-matched antibodies (PE-Rat IgG2b κ Isotype; APC-Rat IgG2a κ Isotype, Biolegend). Antigen expression was determined using a Becton–Dickinson LSR Fortessa™ multicolor cytometer (Franklin Lakes, NJ, USA). Ten thousand events were recorded for each sample and isotype matched-conjugate. Data were analyzed using FlowJo software and gating for each antibody was determined based on non-specific binding of appropriate negative isotype stained controls [6].

### Western blot analysis

After treatments, cells were washed twice in ice-cold phosphate-buffered saline (PBS) 0.1%, scraped off, and centrifuged at 1500 rpm for 10 min at 4 °C. For protein extraction, cell pellets were homogenized in 100 μL of lysis buffer (1 M TrisHCl pH 7.4, 0.5 M EDTA, 1 M MgCl_2_, 1 M dithiothreitol, 1 M Na orthovanadate, 100 mMNaF, and protease cocktail inhibitor) on ice. After incubation on ice for 30 min, samples were centrifugated for 6 min at 6000 rpm at 4 °C. Protein concentration was assessed by bicinchoninic acid assay (Interchim, Montluçon, France), according to the manufacturer’s instructions. Equal amounts of protein (50 μg) were loaded and separated on SDS-polyacrylamide gels (10%) and transferred onto polyvinyl difluoride membranes (Merck Millipore, Molsheim, France). Membranes were saturated with a blocking solution containing 5% nonfat dried milk and 0.05% Tween-20. Membranes were incubated overnight at 4 °C in a solution containing 5% BSA with the following primary antibodies: anti-phospho-STAT3 (rabbit, Cell Signaling, 1:500), anti-STAT3 (rabbit, Santa cruz, 1:1000), and anti-actin (rabbit, Biolegend, 1:2500). After washing, membranes were incubated for 1 h at room temperature with a rabbit peroxidase-conjugated secondary antibody (1:5000, Jackson Immuno Research Laboratories, Westgrove, PA, USA). The blots were developed using Western Lighting Chemiluminescence Reagent Plus (PerkinElmer Life Science, Waltham, MA, USA). Chemiluminescence was captured by a Syngene detection system and quantified by Gene Tools software (Syngene, Cambridge, UK). Between each revelation, membranes were incubated for 15 min in Re-Blot Plus Strong Antibody Stripping Solution (Merck Millipore, Molsheim, France) according to manufacturer’s instructions in order to remove the previous antibody.

### Lipid analyses

Total lipids from brain structures and BV-2 cell preparations were extracted according to the method of Folch et al. [41] and fatty acids were transmethylated according to the method of Morrison and Smith [42]. Fatty acid methyl esters (FAMEs) were analyzed on a FOCUS GC gas chromatograph (Thermo Electron Corporation) equipped with a split injector and a flame ionization detector. Separation of FAMEs was performed with a BPX70-fused silica capillary column (60 m length × 0.22 mm internal diameter, 0.25 μm film thickness; SGE, Courtaboeuf, France). The hydrogen inlet pressure was 100 kPa. The injector and detector temperatures were at 250 and 280 °C, respectively. The oven was at 150 °C, increased to 190 °C at a rate of 1.5 °C min − ^1^ with a 27-min hold, and increased to 230 °C at a rate of 20 °C min − ^1^ and then left at this temperature for 25 min. FAMEs were identified by making a comparison with commercial standards. Fatty acid composition is expressed as the percentage of total fatty acids.

### Statistical analyses

All data are expressed as means ± standard error of the mean (SEM). Statistical significance between multiple groups was analyzed by two-way ANOVA (treatment × LPS). When two-way ANOVA revealed a significant interaction, significant effects were analyzed by Fisher’s LSD post hoc test. For all other measurements, experimental groups were compared using a one-way ANOVA analysis followed by Tukey’s multiple comparison test. All statistical tests were performed using a critical probability of *p* < 0.05.

## Results

### PC-DHA and AceDoPC attenuate LPS-induced IL-6 mRNA expression in the hippocampus

**Fig. 1:**
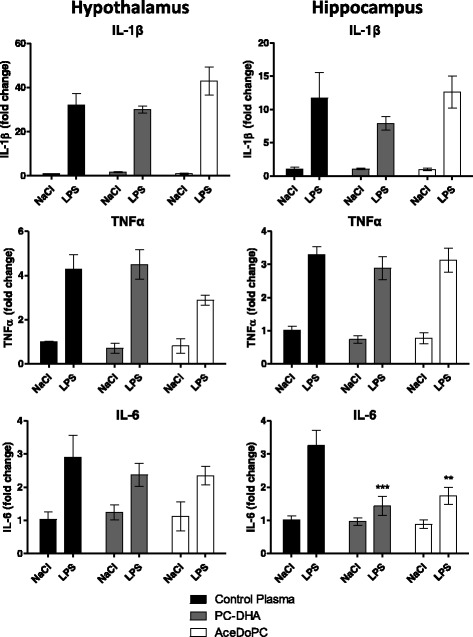
Effect of LPS (6 h, 500 μg/kg, i.p) on cytokine expression in the hypothalamus and hippocampus of mice injected with control plasma (NaCl *n* = 3; LPS *n* = 3), PC-DHA (NaCl *n* = 5; LPS *n* = 6) or AceDoPC (NaCl *n* = 4; LPS *n* = 5). Values are expressed as means ± SEM. **p < 0.01 and ***p < 0.001

To investigate the potential anti-inflammatory effect of PC-DHA and AceDoPC, mice were injected i.v. with PC-DHA or AceDoPC 24 h prior to i.p. LPS administration. LPS is known to induce pro-inflammatory cytokine expression in several brain structures, including the hippocampus and in the hypothalamus [43]. Pro-inflammatory cytokine mRNA expression was measured by RT-qPCR in these two brain structures 6 h after LPS injection (Fig. 1). LPS increased mRNA expression of IL-1β, TNFα, and IL-6 in both the hypothalamus and the hippocampus of the mice (hypothalamus: LPS effect IL-1β F(1, 18) = 110.6, *p* < 0.001; TNFα F(1, 18) = 47.30, *p* < 0.001; IL-6 F(1, 18) = 18.57, *p* < 0.001; hippocampus: LPS effect IL-1β F(1, 22) = 45.25, *p* < 0.001; TNFα F(1, 22) = 81.99, *p* < 0.001). The 2-way ANOVA analysis revealed a significant treatment × LPS interaction (F(2, 22) = 5405 *p* < 0.05). Post-hoc analysis further revealed a significant effect of PC-DHA (*p* < 0.001) and of AcedoPC (*p* < 0.01) on LPS-induced IL-6 mRNA expression.

### PC-DHA and AceDoPC effects on IL-6 mRNA expression in the hippocampus are not associated with changes in plasma IL-6 concentration

**Fig. 2:**
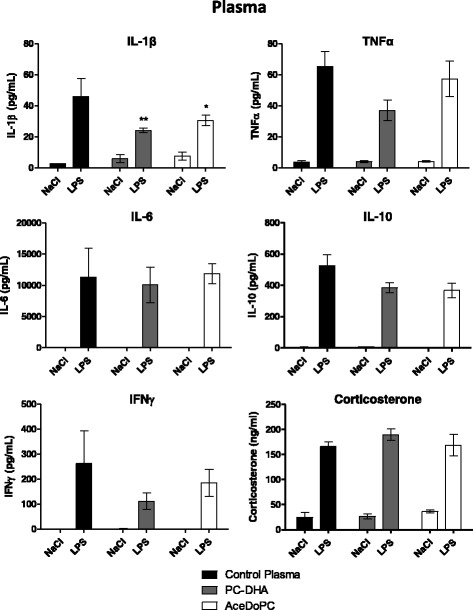
Effect of LPS (6 h, 500 μg/kg, i.p) on cytokine and corticosterone concentration in the plasma of mice injected with control plasma (NaCl *n* = 3; LPS *n* = 3), PC-DHA (NaCl *n* = 5; LPS *n* = 5) or AceDoPC (NaCl *n* = 5; LPS *n* = 4). Values are expressed as means ± SEM. *p < 0.05 and **p < 0.01

To evaluate if PC-DHA and AceDoPC have an effect on peripheral inflammatory response, we then measured the concentration of pro- and anti-inflammatory cytokines in the plasma of mice 6 h after LPS injection (Fig. 2). LPS induced a significant increase in pro-inflammatory and anti-inflammatory cytokine concentration (LPS effect: TNFα F(1, 20) = 85.81, *p* < 0.001; IL-6 F(1, 18) = 31.33, *p* < 0.001; IFNγ F(1, 20) = 24.68, *p* < 0.001; IL-10 F(1, 19) = 260.1, *p* < 0.001) along with an increase in plasma corticosterone levels (LPS effect, F(1, 19) = 213.0, *p* < 0.001). No significant effect of PC-DHA and AceDoPC was observed for TNFα, IL-6, IL-10, IFNγ, and corticosterone levels. Only LPS-induced IL-1β was significantly reduced in mice injected with PC-DHA or AceDoPC in comparison with vehicle-treated mice (interaction treatment × LPS, F(2, 19) = 4.810, *p* < 0.05). Hence, PC-DHA and AceDoPC effect on IL-6 mRNA levels in the hippocampus was not associated with an effect on IL-6 levels at the periphery.

### PC-DHA and AceDoPC effects in the hippocampus are not associated to fatty acid composition changes

**Fig. 3:**
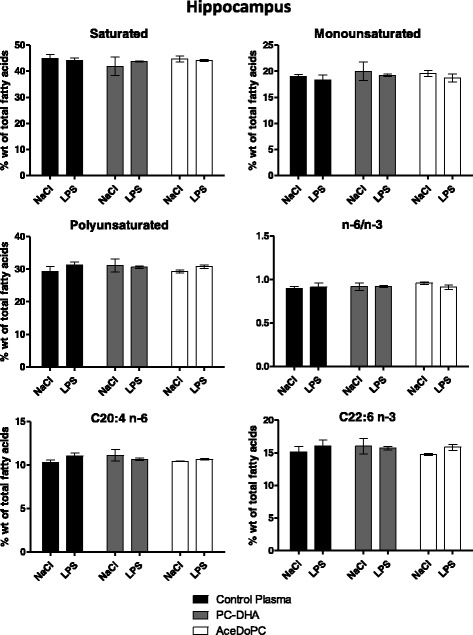
Effect of LPS (6 h, 500 μg/kg, i.p) treatment on fatty acid composition in the hippocampus of mice injected with control plasma (NaCl *n* = 3; LPS *n* = 3), PC-DHA (NaCl *n* = 5; LPS *n* = 6) or Acedo-PC (NaCl *n* = 5; LPS *n* = 6). Values are expressed as means ± SEM. C20:4 n-6: arachidonic acid; C22:6 n-3: docosahexaenoic acid

Fatty acids are known to modulate inflammatory response in the brain, especially in the hippocampus; therefore, we assessed the fatty acid content in this brain structure (Fig. 3). Both PC-DHA and AceDoPC treatments did not change the fatty acid composition in the hippocampus. Moreover, our data showed that 6 h LPS did not alter either the fatty acid composition of this brain structure. Hence, PC-DHA and AceDoPC effects on brain inflammatory response to LPS were not due to changes in hippocampus fatty acid composition.

### Both PC-DHA and AceDoPC attenuate LPS-induced IL-6 mRNA expression in BV-2 cells

**Fig. 4:**
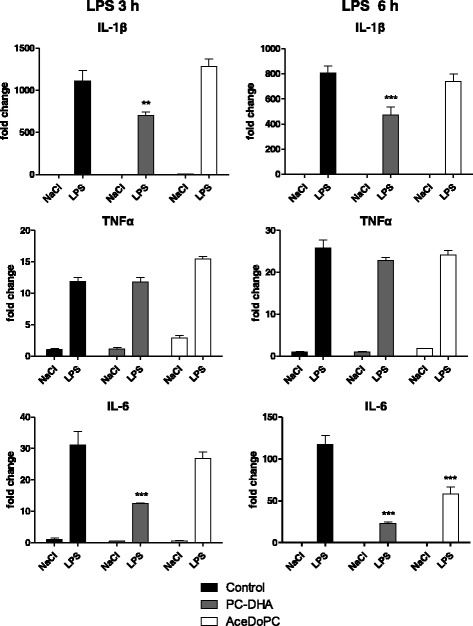
Cytokine expression in BV-2 cells treated with control vehicle, PC-DHA or AceDoPC for 24 h and stimulated with NaCl or LPS for 3 or 6 h. Values are expressed as means ± SEM (*n* = 3). **p < 0.01 and ***p < 0.001

Because PC-DHA and AceDoPC effects in the hippocampus could be due to a direct effect on microglia, the immune cells of the brain, we then assessed their anti-inflammatory effect in vitro, in BV-2 cells. Cells were incubated for 24 h with PC-DHA, AceDoPC, or their control vehicle. LPS-stimulation (3 and 6 h) induced an increase in IL-1β, TNFα, and IL-6 mRNA expression in comparison to NaCl in vehicle-treated cells (Fig. 4). PC-DHA decreased LPS-induced IL-6 mRNA expression 3 and 6 h after stimulation (interaction treatment × LPS: 3 h F(2, 12) = 12.25, *p* < 0.01; 6 h F(2, 12) = 36.88, *p* < 0.001). Moreover, AceDoPC decreased IL-6 mRNA expression 6 h after LPS stimulation in comparison with vehicle group. These effects were specific to IL-6. Indeed, we did not observe any effect of treatments on LPS-induced TNFα mRNA expression and only an effect of PC-DHA on IL-1β mRNA expression 3 and 6 h after LPS stimulation (interaction treatment × LPS: 3 h F(2, 12) = 10.70, *p* < 0.01; 6 h F(2, 12) = 8.75, *p* < 0.01).

### AceDoPC decreases IL-6 receptor subunits surface expression in microglial cells

**Fig. 5:**
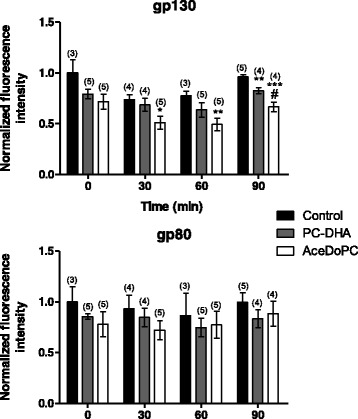
IL-6 receptor subunit protein expression in BV-2 cells treated with control vehicle, PC-DHA or AceDoPC for 24 h and stimulated with IL-6 for 0, 30, 60, and 90 min. Values are expressed as means ± SEM. *p < 0.05, **p < 0.01, and ***p < 0.001 when compared to its respective control group; #*p* < 0.05 when compared to its respective PC-DHA group

We then investigated by flow cytometry analysis whether PC-DHA and AceDoPC exerted their effects on IL-6 expression in BV-2 cells via the IL-6 receptor. We evaluated the BV-2 cell surface expression of gp80 and gp130, two subunits of the IL-6 receptor, after 24 h lipid treatment and 0, 30, 60, or 90 min stimulation with IL-6 (Fig. 5). Lipid treatment and IL-6 did not change the expression of gp80 subunit. However, gp130 expression decreased after AceDoPC treatment (treatment effect: 30 min *F* = 4.072, *p* < 0.05; 60 min *F* = 5.369, *p* < 0.05; 90 min *F* = 17.46, *p* < 0.001). PC-DHA decreased gp130 expression only 90 min after LPS treatment (treatment effect: 90 min *F* = 2.140, *p* < 0.01).

### AceDoPC decreases IL-6-induced STAT3 activation

**Fig. 6:**
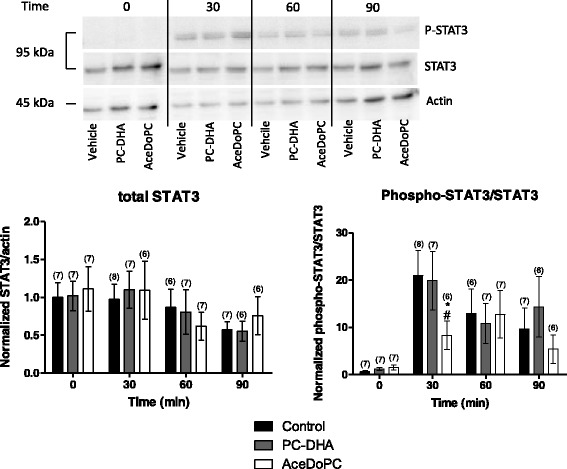
STAT3 protein expression and activation in BV-2 cells treated with control vehicle, PC-DHA or AceDoPC for 24 h and stimulated with IL-6 for 0, 30, 60, and 90 min. Values are expressed as means ± SEM. *p < 0.05 when compared to its respective control group; #*p* < 0.05 when compared to its respective PC-DHA group

Due to the effect of AceDoPC on IL-6 receptor subunit expression, we then assessed if the activation of the pro-inflammatory signaling pathway associated with the IL-6 receptor, the STAT3 pathway, was different under IL-6 stimulation (Fig. 6). When the cells were treated with AceDoPC, 30-min IL-6 treatment decreased STAT3 phosphorylation in comparison to vehicle- and PC-DHA-treated microglial cells (treatment effect: *F* = 3.850, *p* < 0.05). These results suggest that AceDoPC not only had an effect on IL-6 expression but also on its receptor and its associated signaling pathway.

### BV-2 fatty acid composition

Finally, to test whether PC-DHA and AceDoPC affect the fatty acid composition of BV-2 cells, fatty acid contents were assessed by gas chromatography. Table 1 shows that both treatments induced a significant increase in n-3 PUFAs (treatment effect: *F* = 379.6, *p* < 0.001), and specifically in DHA (22:6 n-3) (treatment effect: *F* = 542.8, *p* < 0.001), inducing a decrease in n-6/n-3 ratio (treatment effect: *F* = 118.2, *p* < 0.001). Moreover, data showed that AceDoPC treatment significantly decreased the amount of saturated fatty acids in the membrane of BV-2 cells in comparison with vehicle-treated cells (treatment effect: *F* = 5.218, *p* < 0.05).

**Table 1:** Fatty acid composition of BV-2 cells treated with control vehicle, PC-DHA, or AceDoPC for 24 h. Values are expressed as means ± SEM (*n* = 4)

	Control *n* = 4	PC-DHA *n* = 4	Acedo-PC *n* = 4	*p* value
	mg/100 mg fatty acids			
c12:0	3.1 ± 0.30	1.6 ± 0.79^*^	1.3 ± 0.54^*^	0.01
c14:0	7.1 ± 0.23	5.4 ± 1.48	4.4 ± 1.27^*^	0.05
c15:0	0.4 ± 0.03	0.4 ± 0.01	0.4 ± 0.02	0.46
c16:0	23.9 ± 0.82	21.1 ± 1.83	21.5 ± 1.17	0.07
c17:0	0.3 ± 0.01	0.3 ± 0.01	0.2 ± 0.01	0.54
c18:0	9.3 ± 0.20	9.8 ± 0.43	10.5 ± 0.82	0.07
c20:0	0.5 ± 0.30	0.2 ± 0.01	0.2 ± 0.02^*^	0.04
c22:0	0.3 ± 0.04	0.3 ± 0.06	0.3 ± 0.02	0.13
c24:0	0.9 ± 0.04	1.0 ± 0.09	0.9 ± 0.04	0.25
Saturated	45.6 ± 0.74	40.0 ± 3.56	39.7 ± 2.36^*^	0.04
c16:1n-9	3.0 ± 0.07	3.4 ± 0.22^*^	3.2 ± 0.15	0.04
c16:1n-7	2.5 ± 0.07	2.6 ± 0.12	2.7 ± 0.10	0.08
c18:1 t	2.1 ± 0.36	0.8 ± 0.24^***^	0.6 ± 0.04^***^	< 0.0001
c18:1 n-9	22.3 ± 0.56	23.0 ± 1.40	23.5 ± 0.84	0.40
c18:1 n-7	8.2 ± 0.14	9.1 ± 0.44^*^	9.4 ± 0.44^**^	0.01
c20:1 n-9	0.6 ± 0.01	0.6 ± 0.07	0.6 ± 0.05	0.71
c24:1 n-9	1.3 ± 0.03	1.5 ± 0.11a^*^	1.6 ± 0.10^*^	0.01
Monounsaturated	40.0 ± 0.80	41.0 ± 2.16	41.5 ± 1.53	0.52
c18:2 n-6	0.3 ± 0.02	0.3 ± 0.03	0.3 ± 0.04	0.92
c20:4 n-6	1.4 ± 0.08	1.4 ± 0.10	1.4 ± 0.09	0.86
c22:4 n-6	0.2 ± 0.03	0.1 ± 0.02	0.1 ± 0.01	0.08
c22:5 n-6	0.1 ± 0.02	0.1 ± 0.01	0.1 ± 0.01	0.07
n-6	2.0 ± 0.10	1.9 ± 0.10	1.9 ± 0.11	0.64
c18:3 n-3	0.2 ± 0.01	0.2 ± 0.01	0.2 ± 0.02	0.26
c22:5 n-3	0.3 ± 0.12	0.4 ± 0.22	0.4 ± 0.02	0.43
c22:6 n-3	0.6 ± 0.08	3.7 ± 0.19^***^	3.9 ± 0.11^***^	< 0.0001
n-3	1.1 ± 0.19	4.3 ± 0.21^***^	4.6 ± 0.13^***^	< 0.0001
c20:3 n-9	5.9 ± 0.07	6.3 ± 0.68	5.8 ± 0.44	0.34
Polyunsaturated	9.0 ± 0.32	12.5 ± 0.95^***^	12.3 ± 0.61^***^	< 0.0001
dma16:0	1.9 ± 0.03	2.5 ± 0.15^**^	2.5 ± 0.13^***^	< 0.0001
dma18:0	0.9 ± 0.03	1.0 ± 0.12	1.1 ± 0.10	0.13
dma18:1 n-9	1.4 ± 0.03	1.6 ± 0.13	1.5 ± 0.06	0.07
dma18:1 n-7	1.2 ± 0.02	1.4 ± 0.08^*^	1.4 ± 0.08	0.02
DMA	5.4 ± 0.10	6.5 ± 0.48^*^	6.5 ± 0.35^*^	0.01
n-6/n-3	1.8 ± 0.26	0.4 ± 0.01^***^	0.4 ± 0.01^***^	< 0.0001

*p < 0.05, **p < 0.01, and ***p < 0.001 for comparison with control

*DMA* dimethylacetals, *PC* phosphatidylcholine

## Discussion

In this work, we found that in vivo, a single injection of AceDoPC or PC-DHA decreases LPS-induced IL-6 production in the hippocampus of mice. To further understand whether the effect of these molecules was due to their activity on microglia, we tested their activity in vitro. Our results revealed that both AceDoPC and PC-DHA were able to decrease LPS-induced IL-6 expression, while PC-DHA had also an effect on IL-1β. In addition, these molecules reduced IL-6 receptor surface expression while only AceDoPC decreased IL-6-induced STAT3 phosphorylation. Altogether, these results highlight the potency of AceDoPC to regulate IL-6 production and signaling in microglia.

In the last decade, DHA has been recognized as a molecule with anti-inflammatory activity in the brain [1, 44]. This activity is thought to be linked to its direct [14] or indirect [16] effect on microglia, thereby opening strategies for their use in several brain diseases with an inflammatory component [45]. In rodents, brain DHA increase through dietary, genetically or pharmacologically means protects from neuroinflammation linked to aging [5], pro-inflammatory treatment [3, 14, 46], or acute injury [47–49]. Importantly, the acute increase of DHA in the hippocampus of mice is sufficient to attenuate neuroinflammatory processes triggered by the i.c.v. administration of LPS [4, 9]. Conversely, rodent studies with n-3 PUFA dietary deficiencies leading to decreased DHA brain levels result in increased inflammatory cytokine expression, in particular IL-6 in the brain [7, 8, 50]. In humans, lower levels of blood DHA were associated to higher IL-6 levels and depression/anxiety scores after an interferon treatment or in healthy young adults [51–53]. Interestingly, EPA and DHA supplementation reduce inflammatory markers in depressed subjects [54]. However, whether a single, acute administration of DHA controls neuroinflammation has not been evaluated. Repeated intraperitoneal administration of DHA decreases neuroinflammatory pathways activated by traumatic brain injury in rats [55]. Recent work reports that intravenous administration of unesterified DHA induces a transient increase in plasma DHA [56] with a rapid brain uptake [26]. Importantly, DHA, when consumed as phospholipid (PL) forms, enters the brain more effectively than as triglyceride forms [4, 57]. In addition, if the plasma non-esterified DHA is sufficient to replace the brain DHA pool, the longer half-life of lysoPC-DHA allows for a longer brain exposure to DHA [26]. This is in line with the discovery of the presence of MFSD2A at the BBB, a specific transporter of DHA in the lysoPC form [33]. We tested the effect of AceDoPC that mimics lysoPC [34] on LPS brain inflammatory response and compared it to PC-DHA. In vivo, both forms decreased LPS-induced IL-6 production in the hippocampus, but not in the hypothalamus. This suggests that these molecules equally targeted the hippocampus. However, this could be independent of their brain accumulation, as we could not detect any increase of DHA in the hippocampus (Fig. 3). This is in line with the work of Bazinet’s group, who elegantly demonstrated that the unesterified DHA pool, but not the total phospholipid DHA, is critical to regulate i.c.v. LPS-induced cytokine production in the hippocampus [4, 9]. However, as DHA was only measured in the whole hippocampus, we cannot rule out that DHA accumulates in specific substructures such as the BBB or a specific cell type, such as microglia. Further studies are warranted to elucidate this point. The long lasting effect of AceDoPC and PC-DHA 6 h after LPS administration could be linked to their longer half-life in the plasma and in the brain [26].

We cannot exclude that the anti-inflammatory effect of administered forms of DHA on hippocampal IL-6 is due to their peripheral effect. Indeed, both molecules decreased IL-1β in the plasma of LPS-treated mice, which could influence IL-6 brain level as IL-1β has been shown to be the main cytokine activating IL-6 production in the brain [58]. Of note, crawling monocytes are detected in cerebral vasculature of LPS-treated mice [37] and could be directly targeted by AceDoPC and PC-DHA before entering the brain, therefore producing less cytokine. However, monocyte entry is dependent on TNFα [20, 37] that is not downregulated by AceDoPC or PC-DHA. As only hippocampal, and not hypothalamic IL-6 expression is affected by DHA, together with the fact that the hippocampus is more sensitive to DHA variation [19], we speculate that DHA targets this structure. This direct effect of esterified form of DHA is consistent with previous studies showing that a single injection of DHA reduces microglia activation after spinal cord injury [59, 60] or stroke [13, 61]. However, additional in vivo experiments are warranted to determine whether AceDoPC and PC-DHA enter the hippocampus and target microglia to reduce IL-6 production in the context of LPS administration. In addition, according to our in vitro data showing that both IL-6 production and IL-6 signaling are downregulated by AceDoPC and PC-DHA, the analysis of their effect on IL-6 signaling in the hippocampus is of interest for future studies.

To assess whether AceDoPC and PC-DHA target microglia, which are the main cells producing pro-inflammatory cytokines in the brain in response to inflammatory stimuli, we assessed these cells in in vitro studies. Both molecules reduced LPS-induced IL-6 expression in microglia, with PC-DHA also reducing IL-1β. Of note, while DHA downregulated LPS-induced IL-1β mRNA expression in vitro, it was not the case in vivo. Our previous work showed that DHA downregulated LPS-induced IL-1β release at high (30 μM) but not low concentration (0.3 and 3 μM) [2], suggesting that a certain amount of DHA is required to regulate LPS-induced IL-1β synthesis and release in microglia. Here, we used ≈ 60 μM of DHA in vivo and 30 μM of DHA in vitro. In vitro, such dose is sufficient to increase DHA levels in microglia (Table 1). Whether the 60 μM of DHA is sufficient to target microglia and IL-1β production in vivo remains to be demonstrated.

To our knowledge, this is the first study to test the anti-inflammatory activity of DHA esterified on phospholipids in microglia. Free DHA has been previously reported to reduce LPS-induced IL-1β, IL-6, and TNFα through its effect on LPS receptors and signaling pathway [2]. Other mechanisms such as antioxidant activity [62] or lipid bodies and organelle reorganization [63, 64] have been demonstrated to mediate the anti-inflammatory activity of unesterified DHA. In this study, we identify an additional effect of AceDoPC on IL-6 receptors and STAT3 phosphorylation, which is a hallmark of IL-6 signaling pathway activation. IL-6-induced-STAT3 in the brain has been reported to be important in mediating the behavioral effect of LPS [7]. In addition, this pathway is regulated in vivo by DHA content in the brain [7]. DHA inhibitory effect on IL-6 activity has been previously reported in hepatocytes and osteoblasts, with a very specific effect [65, 66]. Of note, DHA inhibitory effect on STAT3 phosphorylation has been previously reported to involve PPARγ [65, 67]. In human gastric epithelial cells, DHA increased the mRNA level of SOCS3, a negative regulator of STAT3 signaling [25, 67]. Interestingly, bacteria-induced STAT3 activation is reduced by DHA through a specific PPARγ-SOCS3 (a negative regulator of STAT3 signaling) [25, 67]. Whether this is the case in microglia in vitro and in vivo remains to be evaluated.

Previous work suggests that COX-2 upregulates IL-6 associated STAT3 signaling [68]. In an in vitro assay, AceDoPC reduces COX-2 activity (data not shown). In line with this, previous work conducted in macrophages revealed that DHEA, a DHA-derivative ethanolamide, downregulates COX-2 expression [26, 69]. However, whether AceDoPC’s specific effect (direct or through derivatives) on the IL-6 signaling pathway involves COX-2 remains to be tested. An additional mechanism underlying AceDoPC’s effects on IL-6 induced-STAT3 phosphorylation could be linked to the decreased expression of gp130, the co-receptor of IL-6. Recent studies highlight that gp130/STAT3 phosphorylation is involved in the pro-inflammatory activity of IL-6, while gp130 mediates IL-6 effect on tissue repair and recovery after infection [70]. Further studies are warranted to determine whether AceDoPC decreases brain IL-6 activity through its effect on gp130 in vivo.

## Conclusions

These results show that AcedoPC modulates LPS-induced neuroinflammation and IL-6 signaling in microglia. As AcedoPC is a specific carrier of DHA and may increase its brain penetration, it represents a therapeutic application for treating neuroinflammation.
